# Mapping Chronic Disease Risk Factors With ArcGIS Online in Support of COVID-19 Response in Florida

**DOI:** 10.5888/pcd18.200647

**Published:** 2021-04-22

**Authors:** Chris DuClos, John Folsom, Jessica Joiner, Melissa Jordan, Keshia Reid, Marie Bailey, Alyssa Cohen, Karen Freeman, Jennifer Johnson, Katherine McDaniel, Ursula Weiss

**Affiliations:** 1 Florida Department of Health, Tallahassee, Florida

**Figure:**
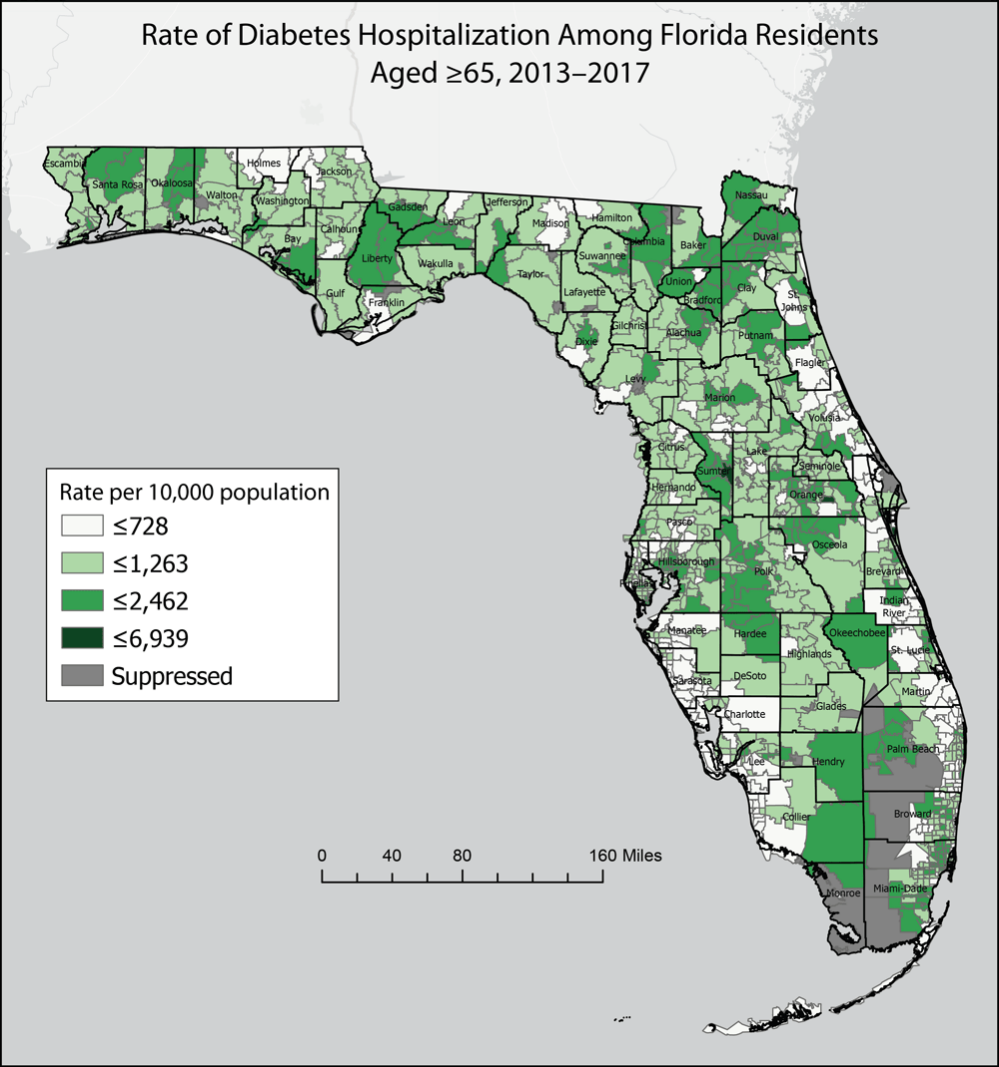
Static display of spatial variability in diabetes, which may increase the risk of severe illness from COVID-19, by Florida zip code and county. In the corresponding interactive map (https://arcg.is/1D0Lz4), 16 measures of chronic disease–related morbidity, mortality, and population health were aggregated to 5-year rates and stratified by age. The map application is used by local county health departments to inform COVID-19 response and identify communities at increased risk of COVID-19. Data on zip codes with <5 health events or <20 residents were suppressed.

## Background

The public health emergency caused by COVID-19 demonstrated that some populations are at increased risk of complications from COVID-19 infection and are at higher risk of death (1). The Florida Department of Health (FDOH) Environmental Public Health Tracking (EPHT) Program and Division of Public Health Statistics and Performance Management collaborated to create a Florida mapping resource to illustrate county and subcounty variability in chronic conditions and related factors that may increase the risk of severe illness from COVID-19. The degree of risk of COVID-19 varies by geography in Florida, and this variability has implications for public health practice as the state seeks to control the pandemic and limit severe health outcomes.

Florida is home to more than 21 million residents from diverse racial/ethnic and cultural backgrounds, approximately 20% of whom are adults aged 65 or older. The proportion of Florida’s older population grew more rapidly than any other age group in the past decade (2). Older adults and people of any age with underlying medical conditions appear to be at higher risk for developing severe illness from COVID-19 than their younger and less medically compromised counterparts (3–5). Cardiovascular conditions, respiratory complications, and diabetes make up a substantial proportion of health conditions and contributing causes in deaths involving COVID-19 (6).

The aims of our mapping project were to describe the spatial variability in populations at particular risk of COVID-19, illustrate patterns of chronic disease at the county and subcounty level, and communicate this information to county health departments and other community members in Florida. Our goal is to reduce severe illness and premature death resulting from COVID-19 among Florida residents and visitors.

We used measures of illness and death resulting from cardiovascular conditions, respiratory conditions, diabetes, and cancer as indicators of increased risk (7–12). We also used county prevalence estimates of chronic disease–related risk factors (tobacco use and obesity) and zip code calculations of economic hardship. We included economic hardship because it is associated with lower rates of health insurance and reduced access to health care services, both of which could lead to COVID-19 complications (5,12).

## Data and Methods

We used 4 data sources for our map. We obtained hospitalization records from the Florida Agency for Health Care Administration to capture data on chronic disease–related morbidity, death data from the FDOH Bureau of Vital Statistics, and data on chronic disease–related risk factors from the 2016 Florida Behavioral Risk Factor Surveillance System (BRFSS) (13). In addition, we calculated the Economic Hardship Index (EHI) by using variables from the 2014–2018 American Community Survey (14). We developed 16 subcounty measures from the hospitalization and death data and aggregated these data to 5-year rates according to zip code. We also mapped the EHI according to zip code. We mapped BRFSS data at the county level, the smallest level of geography available for this data set. We stratified all measures, except EHI, by age to illustrate increases in risk by age. We developed a web-based mapping application in ArcGIS Online and linked this application to existing web data portals at floridatracking.com (the EPHT portal) and FLHealthCHARTS.com. Because we derived the data measures from different sources, we developed a user guide and linked it to the ArcGIS mapping application. This user guide discusses data limitations and the need to interpret data with caution at the subcounty level because of small population sizes. To promote the new mapping application, we added prominent links to the EPHT portal and FLHealthCHARTS.com, which is the most widely used publicly available FDOH data repository and query system (with thousands of visitors per day).

## Highlights

The output of this mapping project is a publicly available online interactive map (https://arcg.is/1D0Lz4) that depicts 5-year trends at the zip code and county levels in Florida. This ArcGIS Online application allows users to easily visualize geographic areas at increased risk of COVID-19. The following indicators of risk of COVID-19 are included on the mapping website: hospitalization and death rates resulting from cardiovascular conditions, respiratory conditions, diabetes, and cancer; the prevalence of tobacco use and obesity; and economic hardship scores.

## Action

The ArcGIS Online interactive map went live in April 2020 and increased access to data measures for chronic conditions and related risk factors that could lead to COVID-19 complications. The use of zip code geography provides for community-level input on populations disproportionately affected by severe outcomes of COVID-19. The mapping application is used by the public and by the state health office and local county health departments to speak with communities about COVID-19 response and to identify areas of Florida at greatest risk for severe outcomes of COVID-19. In addition, the map informed hospital demand planning for the FDOH Bureau of Preparedness and Response, as concerns grew about exceeding hospital bed capacity in Florida. As of February 2021, the map had more than 4,500 views. Other public health programs could use our mapping application as a template for developing a similar application.
